# Mirabegron in patients with age-related macular degeneration treated for overactive bladder: a study protocol for a prospective observational non-randomized trial

**DOI:** 10.3389/fmed.2026.1761473

**Published:** 2026-03-16

**Authors:** Lucia Ambrosio, Maurizio Cammalleri, Serena Panariello, Gianluigi Califano, Arianna Scala, Giovanni Improta, Antonio Pisani, Robert Rejdak, Ireneusz Ostrowski, Luca Filippi, Paola Bagnoli, Massimo Dal Monte, Mario Damiano Toro

**Affiliations:** 1 Department of Public Health, University of Naples Federico II, Naples, Italy; 2 Department of Biology, University of Pisa, Pisa, Italy; 3 Urology Unit, Department of Neurosciences, Reproductive Sciences and Odontostomatology, University of Naples Federico II, Naples, Italy; 4 Department of General Ophthalmology, Medical University of Lublin, Lublin, Poland; 5 Department of Urology and Urological Oncology, Puławy, Poland; 6 Department of Clinical and Experimental Medicine, University of Pisa, Pisa, Italy; 7 Neonatology Unit, Azienda Ospedaliero-Universitaria Pisana, Pisa, Italy; 8 Interdepartmental Center Pisa Neuroscience “PiNeuro”, University of Pisa, Pisa, Italy

**Keywords:** AMD progression, beta 3 adrenoceptor, detrusor muscle, muscarinic receptor, repurposing strategy

## Abstract

Although anti-vascular endothelial growth factor drugs have revolutionized the treatment of neovascular age-related macular degeneration (wet AMD), preventing eyes from converting from dry to wet AMD provides better long-term prognosis for sight and overall health. Mirabegron, an agonist at beta 3 adrenoceptors (β3-ARs), is licensed for the treatment of overactive bladder (OAB), but has potential effects on angiogenic proliferation in the retina, and therefore may reduce risk of conversion from dry to wet AMD. Both OAB and AMD are more common in older adults and share risk factors suggesting a potential link between these two conditions, thus highlighting the need for common therapy for the two diseases. Mirabegron use in AMD patients is supported by its rather safe profile at the eye level as macular and choriocapillary parameters do not seem to be affected in OAB patients. The purpose of this study will be to investigate the effects of mirabegron in patients concomitantly affected by OAB and dry AMD to evaluate its impact on slowing down AMD progression from the dry to the neovascular form.

**Clinical trial registration:**

https://clinicaltrials.gov/study/NCT07305298, identifier NCT07305298.

## 1 Introduction

Sensory impairments, including vision loss, are a part of normal aging that is associated with additional aging-related diseases including those characterized by reduced contractile activity, as in overactive bladder (OAB) wherein urgency and frequent micturition are driven by a multitude of anatomical, metabolic and psychological causes. Among vision impairments proliferative retinopathies including neovascular age-related macular degeneration (wet AMD) pose a significant global burden on individuals, families, and healthcare systems (1). Wet AMD is a major cause of vision loss in elderly people, with an increase in its prevalence in the population over 75 years old. Its estimated increase of about 47% from 2020 to 2040 (2) represents a significant burden to healthcare systems, both in terms of medical treatment and non-medical related costs, such as social services (3). Intravitreal injection of anti-vascular endothelial growth factor (anti-VEGF) is approved for wet AMD. Anti-VEGFs are effective in reducing exudation, but they require frequent injections that, on the other hand, might not be decisive in providing a solution.

Wet AMD is preceded by dry AMD, which is characterized by a gradual degeneration of macular cells and the presence of lipid deposits beneath the retina both contributing to central vision loss. According to the global percentage of dry AMD progression to wet AMD, around 10–15% of patients with dry AMD progress to the wet form (4). Although there is no cure for dry AMD, the pipeline of new treatments to slow its progression is expanding. Once wet AMD is established, anti-VEGF therapy is intended to manage rather than cure wet AMD with most patients experiencing sight loss over time despite regular treatment. Preventing conversion, therefore, has better prognostic value for sight. A number of modifiable risk factors have been found to be effective in slowing AMD progression, such as smoking cessation, and dietary and nutraceutical supplements. In this respect, nutraceutical treatments might limit eye disease progression (5, 6). Among them resveratrol has emerged as a promising agent for mitigating retinal damage (7, 8). Some therapeutic agents have also been licensed for eyes with dry AMD including complement factor inhibitors, which, on the other hand, are only available for individuals with late-stage dry AMD (9). There exists a need for drugs for earlier stages of dry AMD that effectively reduce the risk of progression. Targeting oxidative stress and chronic inflammation might represent an effective treatment as their complex interplay plays a key role in retinal diseases (10).

Potential concomitance between AMD and OAB gave rise to the possibility of investigating whether a therapy that efficiently counteracts OAB may also act on dry AMD progression to wet AMD. As for wet AMD, the global prevalence of OAB is expected to increase by 10.6% from 2020 to 2030 with an increasing trend depending on obesity, gender and age (8). While anticholinergics have been the cornerstone of OAB treatment for decades, their use has consistently been challenged by issues related to side effects and lack of bladder selectivity (11). In particular, there is indication that eye diseases including glaucoma (12) and dry eye disease (13) might occur as side effects of anticholinergic drug administration in OAB patients.

Mirabegron targeting the beta 3 adrenoceptor (β3-AR) is a commonly used drug for OAB, with similar efficacy but fewer side effects than anticholinergic drug options (14). Compared to anticholinergic drugs, mirabegron improves storage function without affecting voiding, thus increasing its therapeutic effectiveness.

In proliferative retinopathies, preclinical data suggest that β3-ARs are involved in retinal vasodilation and are upregulated in ischemic conditions (15). Therefore, potentiating the effect of β3-AR through drug options may be protective against retinal proliferative disorders (such as wet AMD or diabetic retinopathy). Although no human evidence currently exists to support the biological effect of mirabegron on dry AMD progression, in recent studies in OAB patients with healthy eyes, treatment with mirabegron has been demonstrated to be well tolerated at the eye level (16). In addition, mirabegron has been found to increase choroidal thickness as an index of its potential efficacy on choroidal vascularity (17). Overall, these findings encouraged us to follow the path of repurposing mirabegron in patients with AMD treated for OAB to possibly prevent dry AMD progression to wet AMD.

In the present study we hypothesized that the use of mirabegron for concomitant diseases is associated with a reduced risk of progression of AMD. To this aim, we will investigate the effects of mirabegron in patients concomitantly affected by OAB and dry AMD to evaluate its impact on slowing down AMD progression. The potential efficacy of mirabegron on AMD progression will be compared with that of solifenacin, an anticholinergic medication conventionally used to treat OAB. In this respect, the effects of anti-cholinergic drugs in healthy eyes of OAB patients are controversial with a reduction of intraocular pressure by solifenacin as reported by Olcucu et al. (18) although no effects were previously determined by Sekeroglu et al. (19).

## 2 Materials and methods

### 2.1 Study design and participants: inclusion, exclusion and drop out criteria

We planned an observational double arm non-randomized study in two centers to compare the safety and efficacy of either mirabegron (50 mg/day) or solifenacin (5 mg/day) in OAB patients. The protocol provides that ophthalmologists will be blinded regarding which patients have been treated with both drugs. We planned to enroll 312 subjects aged between 50 and 80 years with a diagnosis of early or moderate dry AMD, according to ARDS classification, and OAB. The patients will be enrolled at the Department of Ophthalmology of the University of Lublin, Poland and the University of Naples Federico II, Italy from December 2025 to October 2026. All patients will be followed for a 12-month period. The study will be conducted in compliance with the tenets of the Declaration of Helsinki, accepted by the local Ethics Committee, protocol numbers 2025a45673 at the University of Naples Federico II, Italy and KE-0379/97/2025 at the University of Lublin, Poland.

All study participants will provide written informed consent prior to enrollment and for the use of their clinical data for scientific research in an anonymized manner. Inclusion and exclusion criteria are summarized in Table 1.

**Table 1:** Inclusion and exclusion criteria.

Inclusion criteria
Age 50–80-year-old
Patients with early or moderate dry AMD in one or both eyes
Patients with wet AMD in the other eye
OAB treated for at least 6 months with either mirabegron or solifenacin
BCVA > 65 EDTRS letters
Exclusion criteria
Any contraindication to β-agonists or anticholinergics
Allergy to β-agonists or anticholinergics
Uncontrolled hypertension; tachycardia; atrial fibrillation; long QT or concomitant treatment with drugs that cause lengthening of the QT interval
Patients exposed to experimental drugs other than mirabegron or solifenacin, complement factor inhibitors, photobiomodulation therapy, transscleral lutein iontophoresis therapy or recent surgery (e.g., 3 months)
Recurrent urinary infections
Renal or hepatic failure
Signs of ocular inflammation
Any other ophthalmic diseases such as dry eye, glaucoma, acute or chronic uveitis, advanced cataract
Pregnancy

AMD, age-related macular degeneration; OAB, overactive bladder; BCVA, best corrected visual acuity; EDTRS, early treatment diabetic retinopathy study; QT interval, the time from the start of the Q wave to the end of the T wave in an electrocardiogram.

The unit of analysis will be the eye. When both eyes meet eligibility criteria, both eyes will be included. The study will stop if severe hypertension, tachycardia, or atrial fibrillation develops; in this case, the opportunity to reduce the dose of mirabegron may be considered. The patient will have the right to withdraw their consent at any point during the study. Mirabegron displays no significant increase in major adverse cardiovascular events with a cardiovascular profile almost comparable to that of antimuscarinic agents. In our study, the safe profile of a 50 mg dosage will be associated with an increase of approximately 1 bpm for heart rate and 0.4–0.6 mmHg for systolic/diastolic blood pressure.

### 2.2 Study procedures and outcomes: duration of the treatment and follow up

In order to compare the proportions of solifenacin and mirabegron groups, the estimated sample size for each group has been calculated as 156 participants considering a normal distribution, an *α* error of 0.05 and a power of 80 percent. To evaluate mirabegron safety, cardiac and respiratory parameters (heart rate, blood pressure, oxygen saturation, respiratory support) will be monitored during every study visit. After obtaining informed consent and after the screening visit, at Baseline (T0) all patients will be evaluated with a complete ophthalmic examination. BCVA will be assessed based on the refractive values obtained using an autorefractometer (Huvitz autoref/keratometer HRK-1 Huvitz Co. Ltd., Republic of Korea), in agreement with Early Treatment Diabetic Retinopathy Study (ETDRS) charts. Intraocular pressure measurements will be acquired using the same device. Additionally, slit lamp examination and fundus examination by indirect ophthalmoscopy will be performed in all patients. As part of the imaging protocol, all patients will be evaluated using spectral domain optical coherence tomography (SD-OCT) and fundus autofluorescence (FAF) (SD-OCT Spectralis, Heidelberg Engineering, Heidelberg, Germany). Microperimetry will be performed using MP-1 microperimetry (Nidek Instruments Inc., Padova, Italy). All patients will be evaluated at T0, 6- and 12-months follow-up, with a complete ophthalmic examination, including SD-OCT, FAF, and microperimetry, and monitoring for any signs of ocular inflammation and/or any adverse events.

#### 2.2.1 Primary outcomes

Primary outcomes include conversion to wet AMD at 12 months (assessed by the presence of intraretinal fluid, subretinal fluid, pigment epithelial detachment, subretinal hyperreflactiva material) and outer retinal morphology using SD-OCT and FAF, including reticular pseudodrusen (RPD) type, outer nuclear layer (ONL) thickness, choroidal thickness (ChT), central macular thickness (CMT) and FAF changes.

#### 2.2.2 Secondary outcomes

- Mean BCVA according to the ETDRS charts.
- Mean macular sensitivity (MS).

#### 2.2.3 Safety parameters

Cardiac and respiratory parameters (heart frequency, blood pressure, oxygen saturation, respiratory support).

### 2.3 Data analysis plan and statistical analysis

The sample size was estimated based on epidemiological data and previous literature on AMD progression using planning assumptions for 12 months conversion to wet AMD. Eligibility criteria are reported in Table 1; in brief, contralateral wet AMD status (yes/no) will be recorded at baseline given its strong influence on conversion risk. Published evidence indicates that conversion risk depends substantially on fellow-eye status. In high-risk fellow eyes of patients with unilateral wet AMD, Barbazetto et al. (20) reported a 12-month conversion rate of 20.3% in MARINA and 15.9% in ANCHOR studies. In large trial populations with wet AMD in the contralateral eye, fellow-eye analyses reported cumulative incidences of approximately 26.2–32.2% over 96 weeks (21–23). In contrast, in cohorts without wet AMD in the contralateral eye, annual conversion rates are lower; for example, Chakravarthy et al. (24) reported progression to choroidal neovascularization (CNV) of approximately 3.2 per 100 person-years in bilateral early/intermediate AMD, compared with 15.2 per 100 person-years when CNV was present in the fellow eye (25). Therefore, the expected 12-month conversion rate of 12% has been used as a planning value reflecting the anticipated mix of baseline risk strata in our eye-level cohort, rather than a single incidence applicable to all included eyes. Observed conversion will be reported as stratified by the contralateral wet AMD status (yes/no), and the contralateral status will be included as a key prognostic factor in adjusted analyses.

Assuming a 12% progression rate in the solifenacin group and a 3% progression rate in the mirabegron group at 12 months (absolute difference 9%), with *α* = 0.05 (two-sided) and power = 80%, the required sample size is n ≈ 134 participants per group (net, without accounting for dropouts), computed using the standard formula for comparison of two proportions. The choice of the absolute difference of 9% is not based on clinical data as no clinical trials using either solifenacin or mirabegron to verify the rate of dry AMD progression to the wet form have been performed. This absolute difference was selected as a clinically meaningful planning effect size (Number Needed to Treat ≈ 11 over 12 months). Because eligibility and outcomes are defined at the eye level and participants may contribute with one or two eligible eyes, this calculation should be considered a planning approximation; all inferential analyses will account for within-participant correlation using robust standard errors clustered by participants.

To allow for an expected 15% dropout/non-compliance rate, the enrollment target was increased accordingly, yielding n ≈ 157 participants per group (314 total). For reporting and logistical reasons this has been pragmatically rounded to 156 participants per group (312 total); the protocol will state that this figure includes an allowance for approximately 15% attrition.

The sample size was computed according to the classical formula for two independent proportions:


$$n=\frac{\left(Z_{1-\frac{\alpha }{2}}\sqrt{2p(1-p)}+Z_{1-\beta }\sqrt{p_{1}(1-p_{1})+p_{2}{\left(1-p_{2}\right)}^{2}}\right)}{{\left(p_{1}-p_{2}\right)}^{2}}$$

Where *p*_1_ = 0.12, *p*_2_ = 0.03, *p* = (*p*_1_ + *p*_2_)/2, *α* = 0.05, and 1-*β* = 0.80. The resulting net sample size is approximately n ≈ 134 per group; after adjusting for ~15% attrition the enrollment target is *n* ≈ 157per group (rounded for the protocol to 156 per group).

All statistical analyses will be done using Python in the Google Colab cloud computing environment and/or SPSS (version 31). The primary confirmatory timepoint will be 12 months. To control type 1 error across the pre-specified primary endpoints, *p*-values will be adjusted using the Holm procedure (two-sided). Statistical significance will be set at a two-sided α = 0.05. Analyses will be conducted using exposure-defined (as-treated) and protocol-adherent approaches, rather than randomized intention-to-treat terminology. Continuous variables will be summarized as mean ± standard deviation or median with interquartile range, depending on the data distribution assessed by the Shapiro–Wilk test. Categorical variables will be presented as frequencies and percentages. Baseline characteristics will be described and group balance will be assessed using standardized differences, rather than relying on significance testing of baseline differences.

The primary outcome measures include conversion to wet AMD at 12 months, and outer retinal morphology parameters obtained from SD-OCT and FAF, including RPD type, ONL thickness, ChT, CMT, and FAF changes. These will be analyzed using mixed-effects models to account for repeated measures at T0, 6 months, and 12 months, with fixed effects including treatment group, time, and treatment×time interaction. Robust standard errors clustered by participants will be used to account for correlation between eyes from the same participant. Adjustments will be made for baseline BCVA, age, sex, AMD stage, contralateral wet AMD status (yes/no), and other relevant factors. If assumptions of normality are not met, appropriate non-parametric methods or robust modeling approaches will be used.

Secondary outcomes will consist of changes in BCVA (ETDRS letters) and mean MS (microperimetry). These will be analyzed using the same modeling method, with post-hoc pairwise comparisons adjusted for multiple testing using the Bonferroni method. Given the non-randomized design, primary analyses will use a pre-specified adjusted model including key baseline prognostic factors (age, sex, baseline BCVA, AMD stage/severity, comorbidities, baseline symptom severity, and contralateral wet AMD status), and results will be reported as effect estimates with 95% confidence intervals. Safety parameters (heart rate, blood pressure, oxygen saturation, need for respiratory support) will be reported as the number and percentage of patients with pathological values, without correlation to other variables. Adverse event rates will be compared using χ^2^ or Fisher’s exact tests.

**Figure 1:**
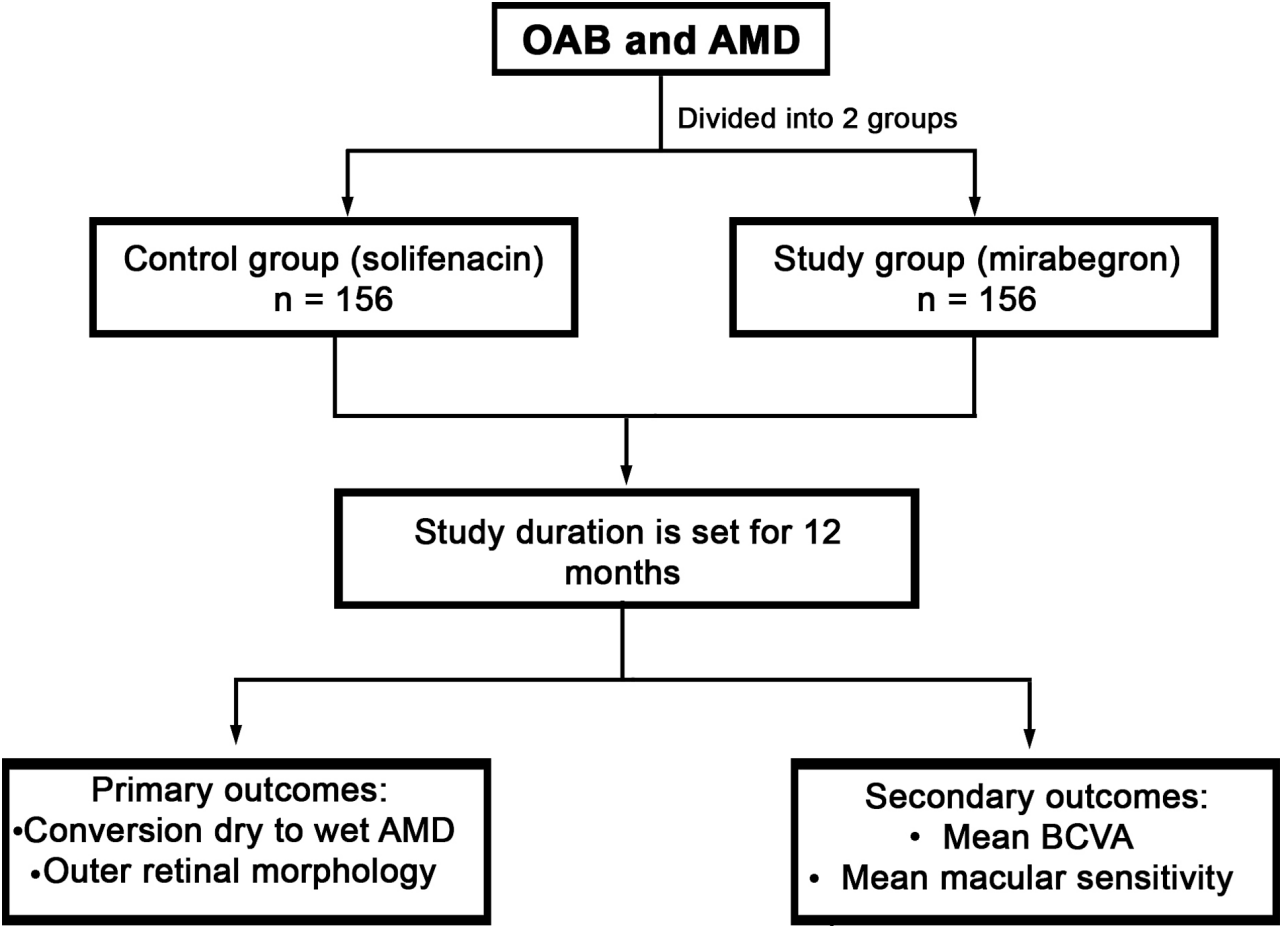
Schematic flow of the proposed trial.

Extreme values will be checked for plausibility and kept unless classified as data entry mistakes or physiologically unlikely. Missing data will be managed according to their missingness pattern, and sensitivity analyses will be conducted to assess the reliability of the results. Exploratory subgroup analyses will be performed according to AMD stage (early vs. moderate). Sensitivity analyses will exclude participants who stop taking mirabegron before 6 months or require a dose adjustment. In Figure 1, trial flow is schematically represented.

### 2.4 Confidentiality

The participants’ data collected during this study will be kept confidential. Study staff will have access to the data as well as the participants’ medical records as they pertain to this study. Published results will not contain any information that would identify individual participants.

## 3 Strengths and limitations

To date, only oral supplementation with antioxidant agents has been proven to slow the progression of dry AMD (26). Recently, the FDA has approved new drugs, the complement inhibitors Avacincaptad Pegol and Pegcetacoplan, for the late stage of dry AMD, but they have not received the approval from the European Medicines Agency. Intervening in the progression of dry AMD by repurposing OAB therapy would be faster and more cost-effective than developing a new drug because the original drug’s safety, dosage, and toxicity profiles are already known. Whether the mirabegron dosage usually used to counteract urinary incontinence in OAB patients might be potentially effective in preventing retinal vessel proliferation is unknown. It is also unknown how long mirabegron should be administered to see any potential effect on dry AMD progression.

## 4 Discussion

Previous exploratory studies in OAB patients failed to demonstrate noticeable effects of mirabegron on visual function. In fact, ocular adverse effects following bladder medication with mirabegron seem to be uncommon and rather dependent on drug dosage, with good tolerability at lower dosages (27). On the other hand, in OAB patients with healthy eyes, mirabegron was found to increase choroidal thickness as an index of its potential efficacy on choroidal vascularity (17). In addition, no significant effects of mirabegron on macular and choriocapillary parameters were recently demonstrated by Cicek et al. (16) in OAB patients without ocular pathologies.

Despite the expanding field of investigation on β3-AR-associated therapies, managing proliferative retinopathies with mirabegron would revolutionize retinal therapy. In this respect, a non-invasive treatment, that is inexpensive, well-tolerated and has few adverse effects would prove to counteract the major burden of eye diseases.

The candidacy of mirabegron as anti-angiogenic drug is based on a number of studies supporting the possibility that dry AMD may be characterized by an early phase of choriocapillaris impairment leading to chronic hypoperfusion activating, in turn, a hypoxic environment that triggers the pro-angiogenic cascade, which culminates in pathological neovascularization thus marking the transition from dry to wet AMD (28–30). In this regard, anti-VEGF treatment, by inhibiting any form of vascularization - both physiological and pathological - may perpetuate the state of retinal hypoperfusion that drives AMD progression. Conversely, treatment with mirabegron might counteract progressive hypoperfusion and therefore reduce or eliminate the need for transition toward the wet form.

**Figure 2:**
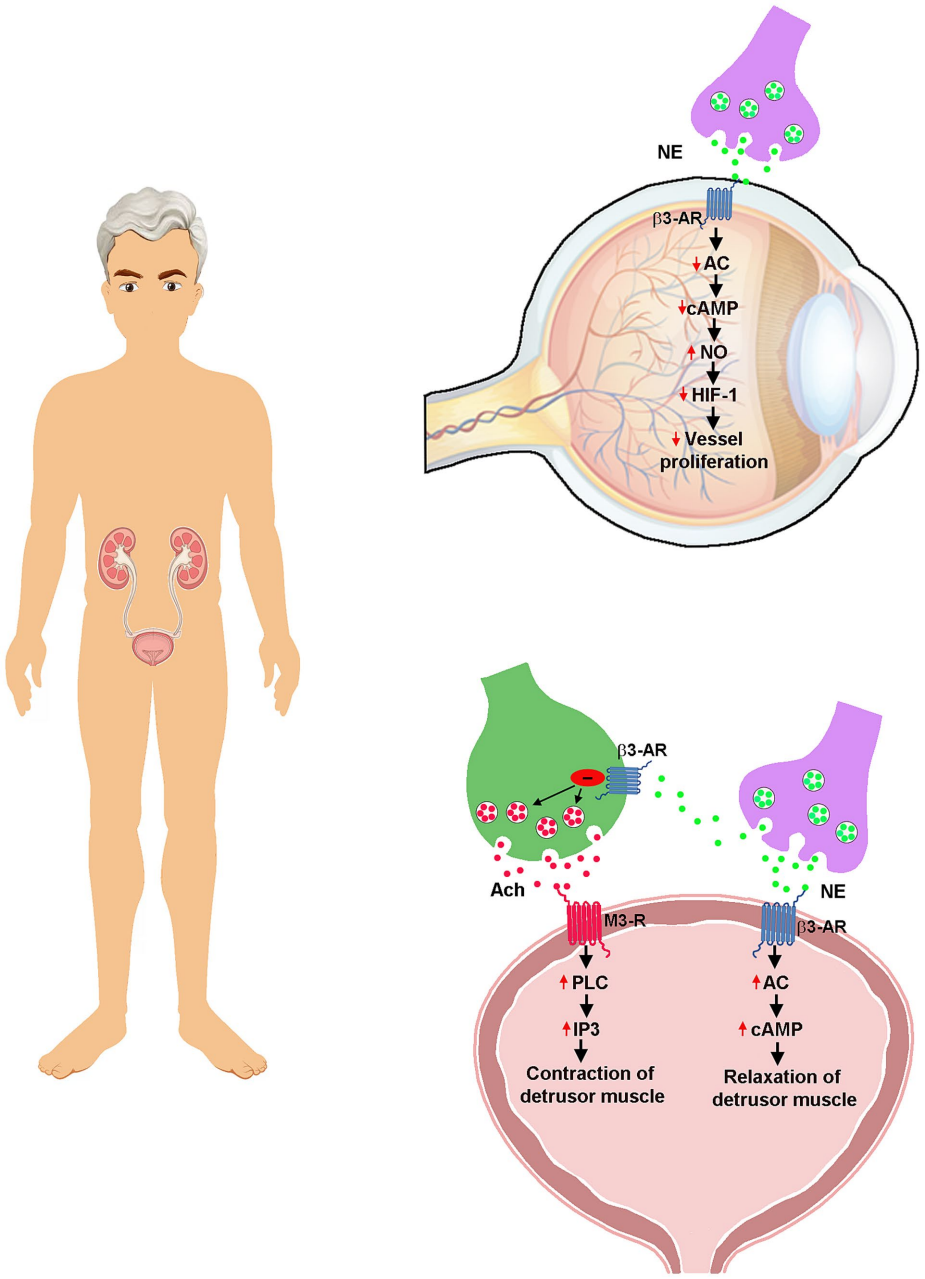
Mirabegron efficacy in the bladder and the retina. In OAB, β3-AR coupling to Gs not only leads to the relaxation of the detrusor muscle through increased levels of cAMP but also acts presynaptically at the level of the parasympathetic nerve terminals to regulate Ach release. Ach coupling to M3-R, through Gq, leads to detrusor muscle contraction by activating PLC that in turn generates IP3 by which Ca^++^ is released by the sarcoplasmic reticulum. In AMD, potential mechanisms mediating reduced vessel proliferation limiting angiogenesis progression include β3-AR activation by NE released after hypoxia-induced overactivation of the sympathetic system. β3-AR coupling to Gi leads to increased NO production that, in turn, prevents HIF-1 dimerization by activating prolyl hydroxilases finally leading to reduced retinal vessel proliferation. AC, adenylyl cyclase; Ach, acetylcholine; AMD, age-related macular degeneration; cAMP, cyclic adenosine monophosphate; Gq, phospholipase C-stimulating G protein; Gs, adenylate cyclase-stimulating G protein; HIF-1, hypoxia-inducible factor 1; IP3, inositol trisphosphate; M3-R, muscarinic acetylcholine receptor M3; NE, norepinephrine; NO, nitric oxide; OAB, overactive bladder; PLC, phospholipase C; β3-AR, β3 adrenoceptor.

In this respect, our preliminary findings in the model of oxygen-induced retinopathy suggest a major effect of β3-AR in inhibiting proangiogenic signaling and favoring endothelial cell homeostasis by activating nitric oxide bioavailability through β3-AR coupling to the nitric oxide synthase pathway (15). Considering that β3-AR activation triggers a cascade of events that, in the bladder, leads to detrusor muscle relaxation while, in the retina, plays a pèotential role in regulating neovessel proliferation in response to an ischemic insult, we hypothesized that β3-AR stimulation with mirabegron could be useful to prevent dry AMD from transitioning to wet AMD by impeding the development of leaky new blood vessels in the macula (Figure 2).

This study will provide data to determine if mirabegron, used in patients with OAB and concurrent AMD, can safely and effectively reduce the conversion of eyes from dry to wet AMD. Any favorable results of this research could open new perspectives and original scenarios to verify the role of off-label mirabegron in preventing neovascular diseases of the eye.

## References

[1] Hariprasad SM Holz FG Asche CV Issa A Mora O Keady S . Clinical and socioeconomic burden of retinal diseases: can biosimilars add value? A narrative review. Ophthalmol Ther. (2025) 14:621–41. doi: 10.1007/s40123-025-01104-3, 40009268 PMC11920568

[2] Wong WL Su X Li X Cheung CM Klein R Cheng CY . Global prevalence of age-related macular degeneration and disease burden projection for 2020 and 2040: a systematic review and meta-analysis. Lancet Glob Health. (2014) 2:e106–16. doi: 10.1016/S2214-109X(13)70145-1, 25104651

[3] Cruess AF Zlateva G Xu X Soubrane G Pauleikhoff D Lotery A . Economic burden of bilateral neovascular age-related macular degeneration: multi-country observational study. PharmacoEconomics. (2008) 26:57–73. doi: 10.2165/00019053-200826010-00006, 18088159

[4] Abdulrazzaq GM Merkhan MM Billa N Alany RG Amoaku WM Tint NL . Current and emerging therapies for dry and neovascular age-related macular degeneration. Pharm Dev Technol. (2025) 30:1147–82. doi: 10.1080/10837450.2025.2562196, 40970447

[5] Bucolo C Musumeci M Salomone S Romano GL Leggio GM Gagliano C . Effects of topical fucosyl-lactose, a milk oligosaccharide, on dry eye model: an example of nutraceutical candidate. Front Pharmacol. (2015) 6:280. doi: 10.3389/fphar.2015.00280, 26635610 PMC4649014

[6] Marchesi N Capierri M Pascale A Barbieri A. Different therapeutic approaches for dry and wet AMD. Int J Mol Sci. (2024) 25:13053. doi: 10.3390/ijms252313053, PMC11641571 39684764

[7] Godos J Romano GL Gozzo L Laudani S Paladino N Dominguez Azpíroz I . Resveratrol and vascular health: evidence from clinical studies and mechanisms of actions related to its metabolites produced by gut microbiota. Front Pharmacol. (2024) 15:1368949. doi: 10.3389/fphar.2024.1368949, 38562461 PMC10982351

[8] Zhang L Cai N Mo L Tian X Liu H Yu B. Global prevalence of overactive bladder: a systematic review and meta-analysis. Int Urogynecol J. (2025) 36:1547–66. doi: 10.1007/s00192-024-06029-2, 39951109 PMC12464077

[9] Lazzara F Conti F Giuffrida E Eandi CM Drago F Platania CBM . Integrating network pharmacology: the next-generation approach in ocular drug discovery. Curr Opin Pharmacol. (2024) 74:102425. doi: 10.1016/j.coph.2023.102425, 38183849

[10] Amato R Lazzara F Chou TH Romano GL Cammalleri M Dal Monte M . Diabetes exacerbates the intraocular pressure-independent retinal ganglion cells degeneration in the DBA/2J model of Glaucoma. Invest Ophthalmol Vis Sci. (2021) 62:9. doi: 10.1167/iovs.62.9.9, 34232257 PMC8267218

[11] Ochoa DC Bouchard B Abrams P. A historical perspective on anticholinergics in overactive bladder (OAB) treatment: “foundations, current practices, and future prospects”. Continence. (2024) 12:101707. doi: 10.1016/j.cont.2024.101707

[12] Eskandar OS Eckford SD Whittaker KW. Treatment of overactive bladder (OBA) with anti-cholinergic drugs and the risk of glaucoma. J Obstet Gynaecol. (2005) 25:419–21. doi: 10.1080/01443610500160675, 16183571

[13] Katipoğlu Z Abay RN. The relationship between dry eye disease and anticholinergic burden. Eye (Lond). (2023) 37:2921–5. doi: 10.1038/s41433-023-02442-x, 36759707 PMC10517132

[14] Jensen CG Secher C Hvid NK Lund L. The history of the pharmacologic treatment of urgency incontinence. Cont Rep. (2024) 11:100059. doi: 10.1016/j.contre.2024.100059

[15] Cammalleri M Amato R Dal Monte M Filippi L Bagnoli P. The β3 adrenoceptor in proliferative retinopathies: "Cinderella" steps out of its family shadow. Pharmacol Res. (2023) 190:106713. doi: 10.1016/j.phrs.2023.106713, 36863427

[16] Cicek MF Ağın A Yakut B Baytaroglu A Gelmis M Ozgor F . Analyzing the impact of a new β3 adrenergic agonist on chorioretinal and peripapillary vessel density. Neurourol Urodyn. (2025) 44:1503–11. doi: 10.1002/nau.70108, 40566846

[17] Topcuoglu M Aslan F. Evaluation of the effect of a novel β3-adrenergic agonist on choroidal vascularity. Invest Ophthalmol Vis Sci. (2021) 62:17. doi: 10.1167/iovs.62.9.17, 34241623 PMC8287045

[18] Olcucu MT Teke K Yildirim K Toğac M Işık B Yilmaz YC. Comparision effects of solifenacin, darifenacin, propiverine on ocular parameters in eyes: a prospective study. Int Braz J Urol. (2020) 46:185–93. doi: 10.1590/S1677-5538.IBJU.2019.0094, 32022506 PMC7025839

[19] Sekeroglu MA Hekimoglu E Petricli IS Tasci Y Dolen I Arslan U. The effect of oral solifenacin succinate treatment on intraocular pressure: glaucoma paradox during overactive bladder treatment. Int Urogynecol J. (2014) 25:1479–82. doi: 10.1007/s00192-014-2396-8, 24803216

[20] Barbazetto IA Saroj N Shapiro H Wong P Ho AC Freund KB. Incidence of new choroidal neovascularization in fellow eyes of patients treated in the MARINA and ANCHOR trials. Am J Ophthalmol. (2010) 149:939–946.e1. doi: 10.1016/j.ajo.2010.01.007, 20378094

[21] Avery RL Castellarin AA Steinle NC Dhoot DS Pieramici DJ See R . Systemic pharmacokinetics and pharmacodynamics of intravitreal aflibercept, bevacizumab, and ranibizumab. Retina. (2017) 37:1847–58. doi: 10.1097/IAE.0000000000001493, 28106709 PMC5642319

[22] Maguire MG Daniel E Shah AR Grunwald JE Hagstrom SA Avery RL . Incidence of choroidal neovascularization in the fellow eye in the comparison of age-related macular degeneration treatments trials. Ophthalmology. (2013) 120:2035–41. doi: 10.1016/j.ophtha.2013.03.017, 23706946 PMC3758381

[23] Parikh R Avery RL Saroj N Thompson D Freund KB. Incidence of new choroidal neovascularization in fellow eyes of patients with age-related macular degeneration treated with intravitreal aflibercept or ranibizumab. JAMA Ophthalmol. (2019) 137:914–20. doi: 10.1001/jamaophthalmol.2019.1947, 31294771 PMC6624808

[24] Chakravarthy U Bailey CC Scanlon PH McKibbin M Khan RS Mahmood S . Progression from early/intermediate to advanced forms of age-related macular degeneration in a large UK cohort: rates and risk factors. Ophthalmol Retina. (2020) 4:662–72. doi: 10.1016/j.oret.2020.01.012 32144084

[25] Kim KL Joo K Park SJ Park KH Woo SJ. Progression from intermediate to neovascular age-related macular degeneration according to drusen subtypes: Bundang AMD cohort study report 3. Acta Ophthalmol. (2022) 100:e710–8. doi: 10.1111/aos.14960, 34390191

[26] The Age-Related Eye Disease Study 2 (AREDS2) Research Group. Lutein + zeaxanthin and Omega-3 fatty acids for age-related macular degeneration: the age-related eye disease study 2 (AREDS2) randomized clinical trial. JAMA. (2013) 309:2005–15. doi: 10.1001/jama.2013.4997 23644932

[27] Bassi A Pur DR Chifor A Malvankar-Mehta MS. Ocular adverse effects of bladder medication: a systematic review. Cutan Ocul Toxicol. (2022) 41:129–36. doi: 10.1080/15569527.2022.2052889, 35546446

[28] Flores R Carneiro Â Neri G Fradinho AC Quenderra B Barata MJ . Choroidal vascular impairment in intermediate age-related macular degeneration. Diagnostics (Basel). (2022) 12:1290. doi: 10.3390/diagnostics12051290, 35626445 PMC9141612

[29] Overbey K Romano F Ding X Bennett CF Stettler I Garg I . Choriocapillaris impairment in dry AMD: insights from swept-source OCT angiography and associations with structural biomarkers. Br J Ophthalmol. (2025) 109:1020–7. doi: 10.1136/bjo-2024-326416, 40360203

[30] Savastano MC Fossataro C Carlà MM Fantozzi C Falsini B Savastano A . OCT angiography analysis of choriocapillaris vascular density in different stages of age-related macular degeneration. Front Ophthalmol (Lausanne). (2022) 2:985262. doi: 10.3389/fopht.2022.985262 38983525 PMC11182125

